# Biofilm architecture and dynamics of the oral ecosystem

**DOI:** 10.5114/bta.2024.145259

**Published:** 2024-12-19

**Authors:** Rina Rani Ray

**Affiliations:** Department of Biotechnology, Maulana Abul Kalam Azad University of Technology, Nadia, West Bengal, India School of Life Sciences, Sambalpur University, Burla, Odisha, India

**Keywords:** biofilm architecture, oral biofilm, subgingival plaque, supra gingival plaque

## Abstract

The oral cavity, being a nutritionally enriched environment, has been proven to be an ideal habitat for biofilm development. Various microenvironments, including dental enamel, supra- and subgingival surfaces, salivary fluid, and the dorsal surface of the tongue, harbor diverse microbes. These biofilms typically consist of four major layers. Depending on the food, age, clinical state, and lifestyle of the patient, the microbial growth dynamics in oral biofilm varies significantly. The presence of pathogenic bacteria that disrupt the normal floral composition of the oral cavity can lead to plaque biofilm formation, which is a precursor to various diseases. Noteworthy pathogenic bacteria, such as *Porphyromonas gingivalis*, *Fusobacterium nucleatum*, and *Streptococcus mutans*, often initiate biofilm formation. Undiagnosed and untreated oral biofilm can lead to severe diseases like periodontitis and eventual tooth loss. Therefore, studying the architecture and dynamics of oral biofilms is essential and can be achieved through image analysis and modern technologies, such as AI-enabled technologies and surface topography-adaptive robotic superstructures.

## Introduction

Biofilm formation is a process in which microbial consortia irreversibly adhere to and grow on biotic or abiotic surfaces with the help of extracellular polymers they produce. Although planktonic cells grow faster than biofilm-bound sessile cells, the latter gain protection from antimicrobial agents like antibiotics and host immune responses. Biofilms develop a three-dimensional structure, influenced by factors such as species composition, the substratum’s nature, and the microenvironment’s pH. The number and spatial distribution of microbial species within a biofilm is mainly affected by the bioconversion of substrates by the resident microbes, biomass expansion, and substrate transport via molecular diffusion (Wanner and Gujer, 1986).

Dental caries, a tooth problem caused by untreated and neglected dental biofilm, can lead to diseases like aggressive and chronic periodontitis (Missoum, 2019), and eventually tooth loss (Ray, 2023a). Bacteria aggregate on the tooth surface and become entangled in an exopolysaccharide-rich matrix, initiating the development of these caries. The fermentation of carbohydrates by resident microbes produces sugar moieties that enrich the matrix, causing acidification of the extracellular milieu. This acidification, in turn, facilitates enamel demineralization and gradual tooth decay. Despite the critical role of acidification, little is known about the role of biofilm structure in enamel demineralization (Xiao et al., 2017).

Microorganisms in the oral cavity have a high likelihood of spreading to nearby areas, including epithelial surfaces. Consequently, persistent oral hygiene issues can lead to various chronic and life-threatening disorders. It has been found that within polymicrobial biofilms, there are isolated pockets formed by the intricate network of the exopolysaccharide matrix, each maintaining a distinct microenvironment characterized by specific pH levels. This compartmentalization within the biofilm architecture results in the differential dominance of microbial species, a phenomenon that can be further influenced by the presence of other specific species in close proximity.

The acidic environment within the three-dimensional structure of the oral biofilm is maintained despite the oral cavity being continuously irrigated by large amounts of neutralizing saliva (Bowen et al., 2018). Acidic microenvironments persist within the biofilm, sustained by the growth of acid-producing bacteria in isolated compartments (Xiao et al., 2012). This three-dimensional architecture of the oral biofilm plays a crucial role in the formation of dental caries, which may lead to periodontitis and eventual tooth loss. Furthermore, detecting oral biofilm and monitoring changes in its dynamics using advanced technologies and AI-based tools has become increasingly important. This minireview aims to highlight the clinical significance of the architectural design of oral biofilm and the application of cutting-edge technologies for detecting changes in biofilm dynamics.

### Normal flora of oral ecosystem

The oral ecosystem consists of the oral microbiome, which resides in various anatomic microenvironments within the mouth and its fluid content. The members of the oral microbial community include bacteria, archaea, fungi, protozoa, and viruses. The oral microbiome exists either as free-floating planktonic organisms suspended in saliva or as biofilm-bound cells that form on oral surfaces (Samaranayake et al., 2016).

Saliva, a fluid secreted by the salivary glands into the oral cavity, contains numerous bacteria that are normal residents of dental surfaces, gums, the dorsal surface of the tongue, and the inner lining of the cheek. A wide range of bacterial species are present in saliva, including *Streptococcus mitis*, *S. salivarius*, *Granulicatella adiacens*, *Neisseria flavescens*, *Rothia mucilaginosa*, and *Prevotella melaninogenica*. These bacterial species generally reflect the local conditions of a person’s oral environment rather than any specific clinical situation. It has been concluded that lower phylogenetic diversity and reduced biofilm formation are associated with better oral health (Takeshita et al., 2016).

### Oral biofilm

The oral cavity provides an ideal environment for the growth and biofilm formation of various microbial species due to its humid, nutrient-rich conditions (Ray and Pattnaik, 2023). Rather than being a random mixture of diverse species scattered across the oral surface, microorganisms within biofilms are organized in spatially structured polymicrobial communities (Bowen et al., 2018). The oral microbiome consists of a variety of aggregated microbial combinations of different sizes, shapes, and compositions, alongside free-living cells (Simon-Soro et al., 2022).

### Formation of oral biofilm

Oral microbiomes contain a large number of planktonic bacteria that tend to adhere to surfaces (Deo et al., 2019), leading to the formation of a matrix-enclosed microbial community known as biofilm. This biofilm matrix creates its microenvironment, which varies depending on the host or other natural niches. Typically, a single microbial species or a community of multiple species initially adheres to the surface as single cells, which are then amplified through the addition of other similar cells via quorum sensing. Bacteria in saliva or synovial fluid often aggregate to form a fully developed biofilm with a distinct architectural backbone. However, little is known about how naturally occurring aggregates influence biofilm formation and community expansion.

### Biofilm architecture of the oral cavity and the key microorganisms

Oral microbial biofilms are three-dimensional organized microbial populations adhering to the solid surface of dental enamel, the surface of the root, or dental implants remaining embedded in an exopolysaccharide matrix. The complex structure of biofilm is a defined and composite structure of different microbes. Biofilm architecture takes into account both two-dimensional and three-dimensional aspects, focusing on cellular arrangements and traits such as width, permeability, irregularity, and fragmentation. Bacterial clusters are found on both subgingival and supragingival surfaces, as well as on dental enamel.

Plaque biofilm in the mouth is a primary cause of several diseases, including dental caries (Ray, 2022). Many periodontal pathogens are Gram-negative bacteria from the Cytophaga-Flavobacterium-Bacteroides (CFB) cluster, which includes various species of *Porphyromonas*, such as *P. gingivalis*, *P. endodontalis*, *P. intermedia*, and *P. nigrescens*. The majority of CFB cluster cells are evenly dispersed in the upper and middle layers of the biofilm. *Prevotella* species, however, colonize the biofilm in microcolonies located on the surface of the biofilm. *P. intermedia*, *P. gingivalis*, and *P. endodontalis* are often observed in microcolonies within the top layer. Additionally, *Parvimonas micra*, a Gram-positive bacterium known to cause periodontitis, can also be found in these top-layer microcolonies. Pathogenic microorganisms tend to be more prevalent in the microcolonies found at the top of the subgingival biofilm.

### Biofilm in saliva

Saliva is a complex biological fluid, consisting of approximately 99% water, along with organic and inorganic ions, peptides, proteins, glycoproteins, and several free amino acids. The major cations in saliva include sodium and potassium, while ammonium, calcium, and magnesium are also present. The most abundant anions are chloride and phosphate, followed by nitrate, nitrite, sulfate, and thiosulfate. Organic anions such as lactate, acetate, propionate, and formate are also found. These components make saliva an ideal environment for biofilm formation. Microorganisms are typically found in clusters within oral fluid, forming a complex community of aerobes and anaerobes, including bacteria previously thought to be early and late colonizers. These microbial aggregates quickly spread and develop into three-dimensional structures, influencing population growth, spatial organization, and community structure. Most of these aggregates evolve into structured biofilm communities (Simon-Soro et al., 2022). The core operational taxonomic units (OTUs) include bacteria such as *Streptococcus salivarius*, *S. mitis*, *P. gingivalis*, *Prevotella intermedia*, *P. melaninogenica*, *Granulicatella adiacens*, *Neisseria flavescens*, *Rothia mucilaginosa*, *Treponema denticola*, *Fusobacterium nucleatum*, *Tannerella forsythia*, *Filifactor alocis*, and cariogenic pathogens like *Streptococcus mutans* (Takeshita et al., 2016).

### Biofilm on subgingival plaque

**Fig. 1:**
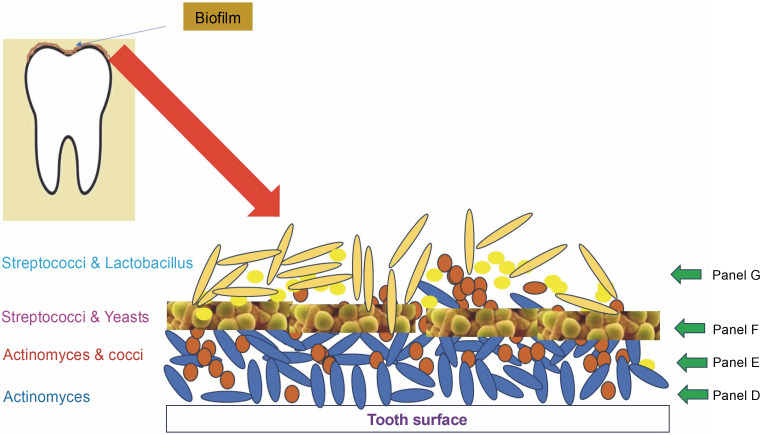
Various strata of a typical biofilm architecture on tooth surface and subgingival plaque.

Subgingival dental plaque, a primary factor in periodontal disorders, is more difficult to study as it is located beneath the gum line. This plaque contains four distinct layers (Fig. 1). The first three layers are found on the tooth surface, embedded within the intercellular matrix, while the fourth layer is loosely organized and lacks clear structure between the attached biofilm and the surrounding soft tissue (Zijnge et al., 2010). The predominant pioneers of biofilm formation on subgingival plaque are species of *Actinomyces* and *Streptococci* (Ximénez Fyvie et al., 2000). It was discovered that the first layer of the biofilm consists solely of *Actinomyces* species.

Pathogenic colonizers such as *Porphyromonas gingivalis*, *Fusobacterium nucleatum*, *Tannerella forsythia*, *Treponema denticola*, and *Aggregatibacter actinomycetemcomitans* form a complex microbial community within just a few days, with the intermediate layer consisting of many spindle-shaped cells of these bacteria. Most of these secondary colonizers, equipped with multivalent adhesins, establish strong bridges between themselves, resulting in a polymicrobial consortium. Potential periodontal infections, such as *P. gingivalis*, can identify and connect to compatible antecedent colonizers, which drives the development of pathogenic subgingival-plaque (Kuboniwa et al., 2000).

The loose fourth layer of subgingival plaque displays a variety of aggregate morphologies. Filaments from the Cytophaga-Flavobacterium-Bacteroides (CFB) cluster, such as *T. forsythia* and *F. nucleatum*, are packed perpendicularly around lactobacilli, creating fine test-tube brush-like structures. These structures are composed of a diverse array of organisms, including *T. forsythia*, *Campylobacter* species, *Parvimonas micra*, *Fusobacteria*, and members of the Synergistetes group A. Synergistetes cells can also form aggregates among themselves.

The uppermost and part of the middle layers of the biofilm predominantly consist of bacteria from the CFB cluster (Zijnge et al., 2010). Ongoing research into coaggregation among subgingival organisms is beginning to uncover the mechanisms behind these relationships.

### Biofilm on supragingival plaque

Biofilms formed on the supragingival region are more heterogeneous in structure compared to subgingival biofilms (Carmello et al., 2020). They are typically divided into two layers: the base layer, which adheres to the dental surface, and an upper layer. The base layer contains biofilms from four distinct microorganisms. The first part consists exclusively of rod-shaped *Actinomyces* cells, followed by a mix of *Actinomyces* species and chains of cocci. The subsequent biofilm is primarily composed of *Streptococcus* and yeasts (Morillo-Lopez et al., 2022).

Corncob structures within supragingival plaque consist of *Streptococcus* species that adhere to the central axis of yeast hyphae (Zijnge et al., 2010). The microbiota of supragingival plaque has been studied using CLASI-FISH (combinatorial labeling and spectral imaging fluorescence in situ hybridization), revealing a spiky “hedgehog”-like radial structure. This structure features radial protrusions of filamentous spines attached to cocci at the periphery, with clusters of cells at the base. Additional cells are found either at the periphery or in an annular ring just below the periphery.

While *Corynebacterium matruchotii* filaments form the framework of this structure, other key participants include *Neisseriaceae*, *Porphyromonas*, *Streptococcus*, *Leptotrichia*, *Fusobacterium*, *Capnocytophaga*, *Actinomyces*, and *Haemophilus-Aggregatibacter*. These “hedgehog” consortia form when *Corynebacterium* cells adhere to a pre-existing biofilm on the tooth surface, providing an attachment surface for *Streptococcus* and *Porphyromonas* cells. These organisms derive nutrients from the crevicular fluid.

In turn, *Streptococcus* cells provide an attachment surface for *Haemophilus* and/or *Aggregatibacter* cells, while creating an anoxic environment in the annulus that promotes the metabolism of *Fusobacterium*, *Leptotrichia*, and *Capnocytophaga* cells. The spatial structure of this consortium is built on a complex network of physical and metabolic relationships. Researchers suggest that *Corynebacterium* species produce filaments from a biofilm base on the tooth surface, which serves as a framework for a multigene metabolic consortium. However, understanding the relative impact of physical and metabolic factors on the spatial arrangement of these organisms is challenging. It is also unclear whether the interactions between species within this biofilm consortium are mutualistic, commensal, or parasitic (Attar et al., 2016).

### Biofilm on dental enamel

Biofilms can grow on human tooth enamel in the presence of sugar, with glucose ingestion rapidly restoring acidic niches, indicating the formation of diffusion barriers associated with the microcolony structure. The demineralization of enamel results in a rougher surface, which enhances bacterial adhesion and promotes biofilm development at the biofilm-enamel interface (Xiao, 2017). Additionally, *Candida albicans* can combine with *S. mutans* and nonmutans streptococci to form a “cross-kingdom corncob” structure on the tooth surface (Kim and Koo, 2020).

### Dynamics of the oral ecosystem

Oral microbiomes can undergo significant changes in composition and activity over time and space, and they are developmentally dynamic in relation to the host. Many factors contribute to these multiplex, nonequilibrium dynamics, including the frequency of host dietary habits, sensitivity to pH variations, microbial interactions, and over time, gene mutations and horizontal gene transfer, which can introduce new traits into bacterial strains.

The oral cavity is a dynamic microbial environment that changes throughout a person’s life. In the first few months of life, the mouth is primarily composed of mucosal surfaces available for microbial colonization. However, with the eruption of teeth, a new hard, nonshedding surface emerges, allowing a greater accumulation of bacteria in the form of biofilm (dental plaque). The gingival crevicular fluid produced in the gingival sulcus provides nutrients for subgingival microorganisms.

As time progresses, the oral ecosystem changes in response to factors such as tooth eruption, extraction, denture implantation, orthodontic band installation, and various dental therapies, including scaling and repair. Temporary shifts in the stability of the oral environment can also be influenced by the frequency and type of dietary intake (Ali et al., 2012).

### Effect of age

In the early stages of life, the absence of teeth limits microbial colonization to the mucosal surfaces, where biofilm formation can occur (Abebe, 2021). As teeth erupt, a larger number of microorganisms begin to accumulate on the hard dental surfaces, forming biofilms and causing dental plaque. As a person ages, lifestyle changes, the onset of diseases, reduced saliva flow, dental treatments such as tooth extraction and insertion of dentures, antibiotic treatment, and other reasons.

### Effect of food

The type of food consumed plays a crucial role in determining the growth of microbial consortia in the oral cavity and, therefore, influences the architecture of oral biofilms. Sugary and cariogenic foods promote the growth of biofilms on dental enamel, which leads to enamel demineralization. This process alters the composition and structure of biofilms, particularly in the development of proximal enamel caries (de Barros Pinto et al., 2019).

### Effect of disease

The composition of biofilms can also change with the onset of various diseases. The relationship between diabetes mellitus and periodontitis is well established, and next-generation sequencing technology has shown that hyperglycemic conditions significantly alter the biodiversity of the subgingival microbiome (Qin et al., 2022). Persons with chronic diseases and compromised general health exhibit changes in the composition of their oral biofilms (Yip et al., 2012).

### Effect of environmental factors and lifestyle

Environmental factors and lifestyle choices, such as smoking, high sugar consumption, pH changes, and the use of certain drugs, can significantly alter biofilm composition. Cigarette smoking and nicotine exposure, for example, intensify the cariogenic activity of oral bacteria, creating a caries-prone environment. Therefore, to maintain good dental health, smokers should quit smoking (Wu et al., 2019). Dietary carbohydrates also enhance oral biofilm formation and contribute to the development of antibiotic resistance, which has become a global public health concern (Jeong et al., 2023).

Sugar fermentation lowers pH, and this acidic environment promotes biofilm formation in the oral cavity. Cariogenic bacteria such as *Streptococcus mutans*, *S. sobrinus*, and *Lactobacillus acidophilus* thrive in acidic conditions, with their metabolic activity and biofilm formation increasing at a pH range of 6-7.5 (Schultze et al., 2021). Consequently, a habit of consuming carbohydrate-rich fast food can adversely affect the biofilm dynamics in the oral cavity.

### Techniques to determine the architecture of oral biofilm

#### Micrograph

Field emission scanning electron microscopy (FESEM) provides high-resolution images that reveal various bacterial cell shapes, allowing for the detection of extracellular material in the form of strands and vesicles (Doherty et al., 2016).

Obtaining an uninterrupted tomography of biofilm structures is challenging because sample collection from clinical areas can easily disrupt the original 3-dimensional (3D) architecture. To overcome this, scientists extracted teeth from patients with severe early childhood caries (ECC), carefully preserving the native biofilm structure. The integrity of the biofilm architecture was evaluated by examining the spatial arrangement of microbial communities on extracted primary teeth from child patients aged 36–72 months with severe ECC. This was achieved through confocal imaging after fluorescent labeling of the bacteria (Kim et al., 2020).

The use of *in vitro* techniques and culture-independent approaches has expanded our understanding of the polymicrobial communities in the oral cavity, as well as the environmental, local, and systemic factors influencing the oral microbiome’s dynamics. Fluorescent in situ hybridization (FISH) enables a thorough understanding of the composition of polymicrobial biofilms (Zijnge et al., 2010), helping to reveal the types of microbial communities present in the oral cavity and the causes of dysbiosis or microbiome disruption. Diseases are the result of such disruptions to homoeostasis. In recent decades, genetic techniques have provided insights into the functional capacity of oral microbiomes, the molecular basis of caries and periodontal disease pathogenesis, and the complex dynamics and fitness determinants of key species within oral microbiomes (Sedghi et al., 2000).

### AI-enabled method for measuring bacterial adherence to dental materials

Dental plaque caused by biofilm has been detected using conventional neural networks (CNN) (You et al., 2020). Recently, scientists have been developing a direct measurement strategy that leverages artificial intelligence (AI) to assess biofilm growth on dental materials through Scanning Electron Microscope (SEM) images. A study discovered that an AI tool, the Fiji Trainable Weka Segmentation (TWS) plug-in, could measure early bacterial adhesion on dental surfaces and detect the presence of bacteria such as *Porphyromonas gingivalis*, *Fusobacterium nucleatum*, and *Streptococcus mutans* (Ding et al., 2022). In another experiment, a U-Net neural network was employed to capture images of teeth, demonstrating reliable detection of dental biofilm (Andrade et al., 2023). Additionally, a deep learning algorithm has been successfully used to predict oral malodor from the salivary microbiome (Nakano et al., 2018).

#### Robotics in oral biofilm detection

Although the use of microrobots for the detection and reduction of oral biofilm in oral healthcare is still in its early stages (Babeer et al., 2022), advancements have been made in computer-assisted detection systems based on deep convolutional neural networks for biofilm detection (Ray et al., 2024). Surface topography-adaptive robotic superstructures (STARS) have also been successfully implemented to diagnose and remove biofilmforming bacteria such as *Streptococcus mutans* (Oh et al., 2022).

## Conclusion

The oral microbiome plays a crucial role not only in maintaining overall health but also in unique personal characteristics, as bacteria, especially those residing in the gums, can identify a person’s ethnicity with the precision of a fingerprint. The normal architecture of oral biofilm remains relatively stable unless disrupted by environmental changes, diseases, or medications. The dynamic growth of oral microbes, influenced by various factors, significantly impacts the onset of oral diseases. This underscores the importance of accurate biofilm detection and analysis in the oral environment.

AI-mediated SMART technologies are increasingly being employed to address these challenges. More technological advancements are being adopted for the successful and accurate detection of biofilm architecture and the altered growth dynamics of biofilm-bound cells.
